# Dual-targeting for brain-specific drug delivery: synthesis and biological evaluation

**DOI:** 10.1080/10717544.2018.1431978

**Published:** 2018-01-31

**Authors:** Qiming Yue, Yao Peng, Yi Zhao, Runxin Lu, Qiuyi Fu, Yang Chen, Yang Yang, Li Hai, Li Guo, Yong Wu

**Affiliations:** Key Laboratory of Drug Targeting and Drug Delivery System of Education Ministry, Department of Medicinal Chemistry, West China School of Pharmacy, Sichuan University, Chengdu, P.R. China

**Keywords:** Glucose, vitamin C, drug delivery, brain targeting, neuroprotective

## Abstract

Ibuprofen is one of the most potent non-steroid anti-inflammatory drugs (NSAIDs) and plays an important role in the treatment of neurodegenerative diseases. However, its poor brain penetration and serious side effects at therapeutic doses, has hindered its further application. Thus, it is of great interest to develop a carrier-mediated transporter (CMT) system that is capable of more efficiently delivering ibuprofen into the brain at smaller doses to treat neurodegenerative diseases. In this study, a dual-mediated ibuprofen prodrug modified by glucose (Glu) and vitamin C (Vc) for central nervous system (CNS) drug delivery was designed and synthesized in order to effectively deliver ibuprofen to brain. Ibuprofen could be released from the prepared prodrugs when incubated with various buffers, mice plasma and brain homogenate. Also, the prodrug showed superior neuroprotective effect *in vitro* and *in vivo* than ibuprofen. Our results suggest that chemical modification of therapeutics with warheads of glucose and Vc represents a promising and efficient strategy for the development of brain-targeting prodrugs by utilizing the endogenous transportation mechanism of the warheads.

## Introduction

Recently, non-steroidal anti-inflammatory drugs (NSAIDs) such as ibuprofen, naproxen and indomethacin have been utilized as neuroprotective agents in treating central nervous system (CNS) diseases (Fan et al., 2011; Zhao et al., 2014; Zheng et al., 2016; Wang et al., 2017). For instance, ibuprofen could reduce the risk or even delay the onset of CNS diseases (Teema et al., 2016), thus would be a promising drug for the treatment of CNS disorders. However, the low brain permeability of ibuprofen would decrease its effective accumulation in the brain and lead to an extremely limited distribution in CNS because of the two physiological barriers that separate the brain from its blood supply. One is the blood–brain barrier (BBB) and the other is the blood–cerebrospinal fluid barrier (BCSFB) (Bernacki et al., 2008; Jakki et al., 2016). The barriers control the entry and exit of endogenous and exogenous compounds. The BBB is maintained by the endothelial tight junctions within the brain microvasculature (Liu et al., 2017; Tian et al., 2017). The BCSFB is located at the choroid plexuses and formed by epithelial cells held together at their apices by tight junctions. Generally, the capability of molecules to cross the barriers by passive diffusion is related to their molecular weight, lipid solubility, charge, hydrogen bonding, ionization profile and physicochemical characteristics. As a result, more than 98% of small molecule drugs and all the macromolecular drugs could not reach the therapeutic concentration in the brain (Saraiva et al., 2016; Zhang et al., 2017). Although increasing the dose of ibuprofen in treatments may increase the vascular-corrected brain concentration, it could cause stronger side effects and severer toxicity. Therefore, there is a huge amount of demand of, not only for ibuprofen but in general, strategies that can effectively deliver drugs, including ibuprofen, into the brain for the treatment of CNS diseases. In this paper, we will explore the possibility to develop a brain-targeting ibuprofen prodrug which uses glucose and vitamin C (Vc) as the mediator to improve the brain permeability.

Because of the high-transport affinity between the transporter and substance, carrier-mediated transporter (CMT) system seems to be one of the most promising methods to facilitate the delivery of drugs into brain. There are plenty of physiological transport systems for nutrients and endogenous compounds, for example, the large neutral amino acid transporter (LAT_1_), monocarboxylic acid transporter (MCT_1_), sodium-dependent vitamin C transporter-2 (SVCT_2_) and glucose transporter (GLUT_1_). GLUT_1_ and SVCT_2_ expressed on the surface of brain capillary endothelial cells and choroid plexus epithelium cells, respectively, are considered as the most efficient transportation systems (Fan et al., 2011; Zhao et al., 2014).

It is well-known that the large and uninterrupted energy demand of the brain is provided almost exclusively by glucose, which is transported through the BBB by GLUT_1_. It is estimated that transport value of GLUT_1_ is 15–3000-fold more than other transporters (Fan et al., 2011). Conjugation of drugs with glucose has been proposed as a strategy to improve their brain uptake (Zhao et al., 2017). Moreover, it has been widely reported that C-6 position glycosylation is an effective way to heighten the accumulation of drugs in brain (Chen et al., 2009). Our previous studies have also shown that the glucose-modified prodrugs can be delivered to the brain specifically and subsequently hydrolyzed to release the parent drugs, hence improving the concentration in the brain (Fan et al., 2011; Zhao et al., 2017). These evidences all suggested that glucose could be used as a good carrier for brain-targeting drugs.

In addition to glucose, it was also reported that the Vc derivatives have a good brain-targeting ability (Wu et al., 2013; Zhao et al., 2014; Qiu et al., 2017). Studies have shown that the concentration of Vc in the brain is the highest, and is 10 times more than that in other organs (Zhao et al., 2014). Its transportation in the brain is mainly through two different ways in general. One is through the sugar transporter GLUT_1_, which can transport dehydro-vitamin C (DHVC, the oxidized form of Vc and can be reduced to Vc in brain). The other way is through the transporter SVCT_2_ which transports Vc into cerebrospinal fluid (CSF) at the choroid plexus, from which Vc can be further diffused to brain extracellular fluid (ECF), and then taken up into the brain cells from ECF. Given the superior ability of Vc in crossing the BBB, it is promising to be used as the carrier for brain-targeting drugs too. Some recent studies also explored the possibility of using Vc as a carrier to improve the BBB permeation and hence to promote the delivery of brain drugs (Pavan et al., 2008). It was reported that the hydroxyl groups of enediol lactone in C2 and C3 are important reaction sites and are required for Vc in the redox process while the C5 and C6-hydroxyl groups of Vc are not critical for its transportation (Zhao et al., 2014).

**Figure 1.**
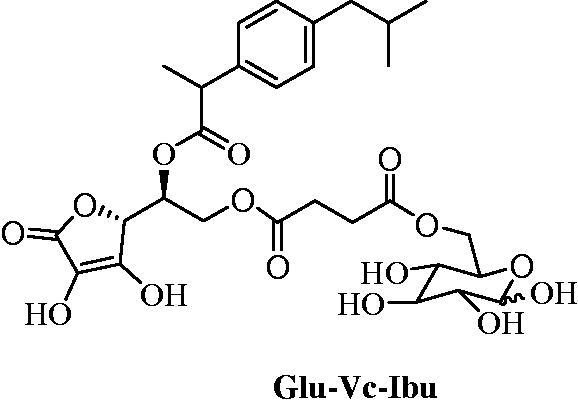
The structure of ibuprofen prodrug Glu-Vc-Ibu.

Our group has been working on brain-targeting drugs for many years. In our previous study, we have studied the glucose or Vc-coupled prodrugs, and demonstrated their brain-targeting ability (Fan et al., 2011; Wu et al., 2013; Zhao et al., 2014; Qiu et al., 2017; Zhao et al., 2017). In this study, we report the design, synthesis and biological evaluation of a dual brain-targeting ibuprofen prodrug (Glu-Vc-Ibu) to further increase the brain permeability (Figure 1). The stability, cell cytotoxicity, neuroprotective effect *in vitro* and *in vivo* of the new prodrug have been studied and compared with the naked ibuprofen, and prodrugs Glu-Ibu and Vc-Ibu.

## Materials and methods

### Materials

Ibuprofen and naproxen were obtained from National Institute for Food and Drug Control. All liquid reagents were distilled before use. The other chemicals and reagents were obtained from commercial sources.

The melting point was measured on a YRT-3 melting point apparatus (Shantou Keyi Instrument & Equipment Co. Ltd., Shantou, China). Infrared (IR) spectra were obtained on a Perkin Elmer983 (Perkin Elmer, Norwalk, CT). ^1^H-NMR spectra were taken on a Varian INOVA 400 (Varian, Palo Alto, CA) using CDCl_3_, dimethylsulfoxide-d6 (DMSO-*d*_6_) and D_2_O as solvent. High-resolution mass spectroscopy data of the product were collected on a Waters Micromass GCT or a Bruker Apex IV FTMS instrument. Reversed-phase chromatography performed on C18 chromatographic analysis was carried out using the high-performance liquid chromatography (HPLC) system (Shimadzu, Kyoto, Japan) consisted of a RF-530 fluorescence detector (Shimadzu) and Allchorom plus data operator, respectively. A Diamonsil column (200 × 4.6 mm, 5 mm) was used. A LC-10A liquid chromatographic system (Shimadzu) and a reverse-phase HPLC column (ODS-C18 column, 4.6 × 200 mm, 5 mm, SinoChrom, Dalian, P.R. China) were used.

### Methods

#### Synthesis of prodrug glu-Vc-ibu

##### Synthesis of compounds 2–9

The synthesis of compounds **2–9** was reported in our previous work (Qu et al., 2014; Zhao et al., 2014).

##### Synthesis of compound 10

To a solution of compound **5** (0.52 g, 0.81 mmol) in CH_2_Cl_2_ (20 ml) was added DCC (0.25 g, 1.22 mmol) and DMAP (20 mg, 0.16 mmol), and the reaction was stirred at −5 °C for 30 min. Then compound **9** (0.31 g, 0.89 mmol) in CH_2_Cl_2_ (10 ml) was added promptly. After stirring for another 6 h at 30 °C, the mixture was filtered and the filtrate was concentrated. Then, the residue was purified by chromatography to give **10** (0.57 g, 88.1%) as a white solid. ^1^H-NMR (400 MHz, CDCl_3_) *δ*: 2.62 (s, 4 H), 3.48–3.58 (m, 3 H), 3.65 (*t*, 1 H, *J* = 8.4 Hz), 4.04–4.05 (m, 1 H), 4.19–4.38 (m, 4 H), 4.49–4.96 (m, 11 H), 5.12 (s, 2 H), 5.14–5.21 (m, 2 H), 7.19–7.37 (m, 30 H). HRMS: (ESI+) calculated for C_58_H_58_O_14_Na [M + Na]^+^ 1001.3724, found 1001.3720. Elemental analysis: C, 71.15; H, 5.97, found C, 71.10; H, 6.03.

##### Synthesis of compound 11

To a solution of ibuprofen (0.16 g, 0.77 mmol) in CH_2_Cl_2_ (15 ml) was added DCC (0.23 g, 1.15 mmol) and DMAP (23 mg, 0.19 mmol), and the reaction was stirred at −5 °C for 30 min. Then, compound **10** (0.37 g, 0.38 mmol) in CH_2_Cl_2_ (10 ml) was added dropwise. After stirring for another 10 h at room temperature, the mixture was filtered and the filtrate was concentrated. Then the residue was purified by chromatography to give **11** (0.34 g, 77.1%) as a white solid. ^1^H-NMR (400 MHz, CDCl_3_) *δ*: 0.89 (d, 6 H, *J* = 6.4 Hz), 1.45 (d, 3 H, *J* = 3.2 Hz), 1.79–1.87 (m, 1 H), 2.16–2.58 (m, 6 H), 3.44–3.86 (m, 6 H), 4.03–5.09 (m, 18 H), 5.34–5.35 (m, 1 H), 6.97–7.38 (m, 34 H). HRMS: (ESI+) calculated for C_71_H_74_O_15_Na [M + Na]^+^ 1189.4925, found 1189.4927. Elemental analysis: C, 73.05; H, 6.39, found C, 73.17; H, 6.30.

##### Synthesis of prodrug Glu-Vc-Ibu

To a solution of compound **11** (0.28 g, 0.24 mmol) in methanol (15 ml), Pd/C (0.17 g, 10%) was added. Then, the mixture was stirred in hydrogen atmosphere at room temperature for 6 h. Pd/C was filtered, and the filtrate was concentrated to give prodrug Glu-Vc-Ibu (0.14 g, 94.0%) as a colorless oil. ^1^H-NMR (400 MHz, CD_3_OD) *δ*: 0.88 (d, 6 H, *J* = 6.4 Hz), 1.42 (d, 3 H, *J* = 7.2 Hz), 1.79–1.86 (m, 1 H), 2.41–2.55 (m, 6 H), 3.11–3.48 (m, 3 H), 3.58–3.72 (m, 2 H), 3.93–3.97 (m, 1 H), 4.14–4.88 (m, 5 H), 5.44–5.46 (m, 1 H), 7.07–7.19 (m, 4 H). HRMS: (ESI+) calculated for C_29_H_38_O_15_Na [M + Na]^+^ 649.2108, found 649.2105. Elemental analysis: C, 55.59; H, 6.11, found C, 55.52; H, 6.17.

#### Synthesis of prodrug Glu-Ibu and Vc-Ibu

The detailed synthetic procedures of prodrug Glu-Ibu and Vc-Ibu were given in the Supplementary material.

#### *In vitro* stability in various buffers

The *in vitro* chemical stability of prodrugs was investigated by incubating the prodrugs in pH 2.5, 5.0, 7.4 and 8.0 phosphate buffers, respectively. Briefly, 1 ml of the prodrugs methanol solution (250 μg/ml) was added into 4 ml different buffer separately. The entire system was kept at 37 ± 0.5 °C with continuous shaking at 100 rpm/min. 200 μl medium samples were taken at the following time points: 0, 0.5, 1, 2, 3, 6, 12, and 24 h, and then replaced by fresh medium. The samples were analyzed by HPLC (Shimadzu). The analysis was accomplished on a SinoChrom ODS-C18 column (Dalian, China) (200 × 4.6 mm, 5 mm), thermostated at 35 °C. The mobile phase was composed of methanol/water (73:27), and the pH was adjusted to 2.86 with 10% phosphoric acid. The UV wavenumber was 225 nm and the flow rate was set at 1.0 ml/min with a 20 μl injection volume.

#### Metabolic stability in plasma and brain homogenate

The preliminary metabolic stability of prodrugs was studied by incubating in plasma and brain homogenate, respectively. 1 ml of the prodrugs methanol solution (250 μM) was added into 4 ml plasma or brain homogenate. Then, the entire system was kept at 37 ± 0.5 °C with continuous shaking at 100 rpm/min. 200 μl samples were taken at the following time points: 0, 0.5, 1, 2, 3, 6, 12, and 24 h, and then replaced by plasma or brain homogenate. The internal standard (naproxen, 10 μl, 30 μg/ml) was added to each sample followed by 200 μl acetonitrile. After centrifuging for 15 min, the supernatants of the samples were analyzed by HPLC method described before.

#### Evaluation of cytotoxicity

The PC12 cells were purchased from the American Type Culture Collection (Rockville, MD). Cells were cultured in DMEM (HyClone Laboratories Inc., South Logan, UT) supplemented with 10% fetal bovine serum (FBS) (Gibco Laboratories, Gaithersburg, MD), 100 U/ml penicillin and streptomycin. Cells were cultured at 37 °C in a 5% CO_2_ humidified environment incubator (Thermo Scientific, Cleveland, OH).

The cytotoxicity of prodrugs and ibuprofen was assayed in PC12 cells using the (3,4,5-dimethylthiazol-yl)-2,5-diphenyl-tetrazolium (MTT) assay. Briefly, PC12 cells were seeded in 96-well plates at a density of 6 × 10^4^ cells/well and cultured for 24 h at 37 °C. The cells were then treated with prodrugs and ibuprofen (10, 20, 50 and 100 μM), respectively and incubated for 24 h. After incubation, 20 μl MTT solution (5.0 mg/ml) was added to the medium and incubated for another 4 h at 37 °C. After removal of the culture medium, the reduced MTT dye was solubi1ized by DMSO (100 μl) and the absorbance was read at 490 nm wavelength on an automatic microplate spectrophotometer. The cell viability = A_490 nm_ for the treated cells/A_490 nm_ for the control cells ×100%.

#### Neuroprotective effect

The *in vitro* neurotoxicity model was established using H_2_O_2_ to induce oxidative stress and injury in PC12 cells. The cell viability, reactive oxygen species (ROS) level and superoxide dismutase (SOD) activity were then determined to measure the protection of ibuprofen and the prodrugs.

To investigate the protective effects of ibuprofen and prodrugs against H_2_O_2_-induced cytotoxicity, the cell viability of PC12 cells was evaluated using the MTT assay. Briefly, PC12 cells were seeded in 96-well plates at the density of 6 × 10^4^ cell/well and incubated at 37 °C for 24 h. Then, the cells were treated with ibuprofen or the prodrugs (10 μM) in FBS-free medium for 4 h and then exposed to H_2_O_2_ (1 μM) for 2 h. After the treatment, the cells were observed under inverted phase contrast microscopy and cell images were recorded. Subsequently, the cells viability was assessed by MTT assay using a microplate reader.

The ROS assay kit (S0033; Beyotime Institute of Biotechnology, Jiangsu, China) in DCFH-DA method was used to determine the intracellular ROS generation. PC12 cells were seeded in six-well plates at a density of 5 × 10^5^ cells/well and incubated at 37 °C for 24 h. After cultured with or without drugs (ibuprofen or prodrugs, 10 μM) in FBS-free medium for 4 h, cells were treated with H_2_O_2_ (1 μM) for 2 h. Then trypsin–EDTA was used to harvest the cells, which were washed three times with PBS afterward, and finally stained with 2′,7′-dichlorofluorescein diacetate (DCFH-DA, 10 μM) and incubated for 20 min at 37 °C in the dark. After centrifugation (2000 rpm, 3 min), the cell pellet was washed three times with PBS, resuspended in PBS before flow cytometer analysis (Ex 488 nm and Em 525 nm). SOD is an index of superoxide level which neutralizes free oxygen radicals, usually being tested along with ROS level showing oxidative stress. In brief, after harvesting, the cells were resuspended in PBS and lysed by Ultrasonic cell grinder (SCIENTZ-II D, Zhejiang, China) to obtain the cell lysis solution. Then, the SOD activity was evaluated using colorimetry kits (A001; Nanjing Jiancheng Bioengineering Institute, Nanjing, China). Untreated cells were used as the control.

#### Brain ischemia model

Adult male Wistar rats weighing 250 ± 20 g were purchased from DOSSY Experimental Animal Center of Chengdu (Sichuan, China). All animal procedures for this study were approved by the Experiment Animal Administrative Committee of Sichuan University (China).

For the *in vivo* study, cerebral ischemia model was induced by bilateral common carotid artery occlusion according to the previously published methods (Wang et al., 2017). All the animals were randomly divided into six groups: sham-operated group, saline group, and the four administration groups (*n* = 5) and received an injection of saline or drugs (1 mg/kg, calculated as ibuprofen) through the tail vein. After 30 min, the animals were anesthetized with 10% chloral hydrate (300 mg/kg, intraperitoneally), tied to the operating table, and exposed the bilateral common carotid artery through a middle incision of the neck and careful separation from the vagal nerves. Then, the arteries were enduringly ligated with bulldog clamps for 1.5 h. The brains were isolated, washed with PBS, frozen at −20 °C for 30 min and cut into 2 mm slices. The brain slices were stained in 1% 2,3,5-triphenyltetrazolium chloride (TTC) at 37 °C for 30 min in dark. Stained sections were photographed with digital camera and the staining rate was calculated by ImageJ (National Institutes of Health, Bethesda, MD).

Furthermore, the remained brain was adequately homogenized with ice-cold saline to determine the levels of SOD, malondialdehyde (MDA) and glutathione (GSH). The activity of SOD, the content of MDA and the level of GSH were measured by using commercial assay kits (Nanjing Jiancheng Bioengineering Institute, Nanjing, China). The tests were performed according to the manufacturer’s instructions.

#### Distribution in brain and pharmacokinetic studies in mice

Kunming mice weighing 20–22 g were purchased from DOSSY Experimental Animal Center of Chengdu (Sichuan, China). All animal procedures for this study were approved by the Experiment Animal Administrative Committee of Sichuan University (P.R. China). The aqueous solutions of ibuprofen and prodrugs (Glu-Vc-Ibu, Glu-Ibu and Vc-Ibu) were prepared with 0.1% Tween-80 to give a final concentration of 1 mg/ml. The mice were injected the ibuprofen or prodrugs solutions (10 mg/kg, calculated as ibuprofen) through the tail vein.

At appropriate time interval (5, 10, 15, 30, 45, 60, 90, 120, 240 and 480 min), blood was collected from the eye socket of mouse into a tube containing heparin, and centrifuged at 5000 rpm for 5 min. The supernatant was collected as plasma sample. The animals were killed by cervical dislocation, and the brain tissue was removed, and then washed with saline for three times to remove the remained blood and rolled over on the filter paper to remove the main vessel. All the brain tissues were homogenized with twice amount of saline. An aliquot of 10 μl of internal standard (naproxen, 30 μg/ml) was added into 200 μl plasma or 200 μl brain homogenate, the mixture was vortexed with 200 μl methanol for 5 min. Then, an aliquot of 50 μl of 6 M NaOH aqueous solution was added to the spiking mixture. After 10 min of hydrolysis at room temperature, ibuprofen was released from the prodrugs, and 50 μl of 6 M HCl was added to neutralize NaOH. Followed by vortexing for 5 min, 0.6 ml acetonitrile was added and the mixture was centrifuged at 15,000 rpm for 10 min. The separated supernatant was evaporated to dryness at 40 °C under air flow. The residues were redissolved in 50 μl methanol and centrifuged at 15,000 rpm for 10 min, and then 20 μl of the supernatant was injected into the HPLC system for analysis. What’s more, the concentrations of released ibuprofen and prodrugs in blood and brain were also analyzed before hydrolysis.

The area under the curve (AUC_0–_*_t_*) and the maximal concentration (*C*_max_) were calculated by Data and Statistics (DAS, Shanghai, China). The statistical analysis of the samples was performed by using a Student’s *t*-test with *p* values < .05 as the minimal level of significance. The relative uptake efficiency (RE) and concentration efficiency (CE) were calculated to evaluate the brain-targeting property of prodrugs. The values of RE_brain_ and CE_brain_ are defined as follows:

$$\text{RE}\;=\;(\text{AUC}{\;}_{0}){\;}_{\text{Pro}}/(\text{AUC}{\;}_{0}){\;}_{\text{Ibu}},$$

$$\text{CE}={\left({\text{C}}_{\text{max}}\right)}_{\text{Pro}}/{\left({\text{C}}_{\text{max}}\right)}_{\text{Ibu}}.$$

#### Statistical analysis

All the data were presented as mean ± standard deviation (SD). Statistical comparisons were performed by analysis of variance (ANOVA) for multiple groups followed by Student’s *t*-test. The threshold for significance was *p* < .05.

## Results and discussion

### Synthesis and characterization of prodrugs

**Scheme 1.**
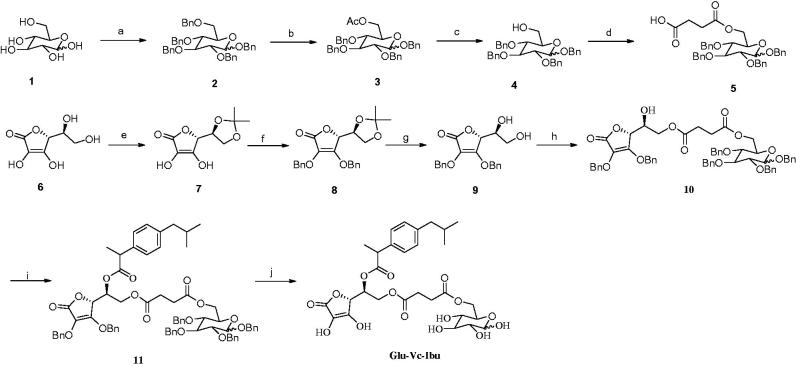
Synthesis of prodrug Glu-Vc-Ibu. Reagents and conditions: (a) NaH, BnBr, DMF, r.t.; (b) Ac_2_O-AcOH, ZnCl_2_, r.t.; (c) CH_3_ONa, CH_3_OH, r.t.; (d) succinic anhydride, DMAP, CH_2_Cl_2_, r.t.; (e) acetone, acetyl chloride, r.t.; (f) BnBr, K_2_CO_3_, acetone, reflux; (g) HCl, CH_3_CN, 30 °C; (h) **8**, DCC, DMAP, CH_2_Cl_2_, r.t.; (i) ibuprofen, DCC, DMAP, CH_2_Cl_2_, r.t.; (j) Pd/C, H_2_, CH_3_OH, r.t.

The synthetic route of prodrug Glu-Vc-Ibu was illustrated in Scheme 1. Firstly, we synthesized the glycosylated derivative **5** and the synthetic route was demonstrated in Scheme 1. Briefly, protection of the hydroxyl groups of glucose **1** with benzyl was furnished by treating with benzyl bromide. Then, selectively acetolysis of the benzyl protecting group at C-6 in compound **2** using ZnCl_2_-Ac_2_O-AcOH gave **3**. Subsequently, deacetylation of **3** under Zemplén condition gave compound **4** in 98.9% yield, which then coupled with succinic anhydride to give acid **5** by using DMAP as catalyst. The synthesis of intermediate **9** started from the available material Vc. Treatment of **6** with acetyl chloride in acetone following our earlier method afforded 5,6-O,O-isopropylidene protected Vc ketol **7** (Zhao et al., 2014). Benzylation of the C-2 and C-3 hydroxy groups of the ketol **7** was accomplished using K_2_CO_3_ and benzyl bromide in acetone to provide **8**. Deblocking of the 5,6-O,O-protected derivative of **8** with HCl in CH_3_CN solution gave 2,3-O,O-dibenzyl Vc intermediate **9**, which coupled with compound **5** in the presence of DCC and DMAP to generate **10** in 88.1% yield. Subsequent esterification of **10** was carried out with the ibuprofen to give compound **11** by using DCC as a condensing agent. Finally, the debenzylation of the benzyl groups with 10% Pd/C reached the prodrug Glu-Vc-Ibu. All the title compounds and important intermediates were characterized by their respective IR, ^1^H-NMR and MS.

### *In vitro* stability in various buffers

In order to investigate the chemical stability of the prodrugs, Glu-Vc-Ibu, Glu-Ibu and Vc-Ibu were incubated in pH 2.5, 5.0, 7.4 and 8.0 phosphate buffers, respectively. These solutions were maintained at 37 °C, and the aliquots were withdrawn in predetermined time intervals, then the concentrations of the prodrugs were determined by HPLC method. The pseudo first order rate constants (K_disapp_) and half-lives (*t*_1/2_) of these prodrugs in aqueous solutions were calculated by linear regression of the peak area against time, and were illustrated in Table 1. It could be seen that all the prodrugs appeared to be highly stable in pH 5.0 buffer solution, moderately stable in pH 2.5 and instable in pH 7.4 and 8.0 buffer solution. According to this result, the slow hydrolysis of the prodrugs ensured that the parent drug ibuprofen could be slowly released and sustained in the physiological environment at pH 7.4.

**Table 1.** Chemical stability of prodrugs at 37 °C.

		Kinetic constants	
Prodrug	pH value	*K*_disapp_ (h^−1^)	*t*_1/2_ (h)
Glu-Vc-Ibu	2.5	3.84 × 10^−2^	18.05
	5.0	1.39 × 10^−2^	49.87
	7.4	5.40 × 10^−2^	12.84
	8.0	6.25 × 10^−2^	11.09
Glu-Ibu	2.5	4.03 × 10^−2^	17.20
	5.0	1.94 × 10^−2^	35.73
	7.4	6.66 × 10^−2^	10.41
	8.0	7.59 × 10^−2^	9.13
Vc-Ibu	2.5	3.13 × 10^−2^	22.15
	5.0	2.19 × 10^−2^	31.65
	7.4	7.69 × 10^−2^	9.01
	8.0	8.63 × 10^−2^	8.03

### Metabolic stability in plasma and brain homogenate

The metabolic stability of the prodrugs was studied in the mice plasma and brain homogenate at 37 °C. The K_disapp_ and *t*_1/2_ of the prodrugs in plasma and brain homogenate were calculated by linear regression of the peak area against time (Table 2). As expected, the three prodrugs showed considerable stability in plasma, where *t*_1/2_ were found to be 61.89, 72.96 and 51.34 min for prodrug Glu-Vc-Ibu, Glu-Ibu and Vc-Ibu, respectively. The long half-life time guaranteed that they had sufficient time to reach the brain before they were metabolized.

**Table 2.** Metabolic stability of prodrugs in mice plasma extracts and brain homogenate at 37 °C.

		Kinetic constants	
Prodrug	Biological matrix	*K*_disapp_ (min^−1^)	*t*_1/2_ (min)
Glu-Vc-Ibu	Plasma	1.12 × 10^−2^	61.89
	Brain	3.25 × 10^−2^	21.33
Glu-Ibu	Plasma	0.95 × 10^−2^	72.96
	Brain	2.62 × 10^−2^	26.46
Vc-Ibu	Plasma	1.35 × 10^−2^	51.34
	Brain	4.16 × 10^−2^	16.66

As shown in Table 2, all the prodrugs had a faster metabolic rate in brain homogenate than in plasma. The *t*_1/2_ of the Glu-Vc-Ibu, Glu-Ibu and Vc-Ibu in brain homogenate were 21.33, 26.46 and 16.66 min, respectively, which indicated that these prodrugs were hydrolyzed rapidly in the brain. It may be attributed to the abundant esterase in brain that could degrade the prodrugs to release the parent drug ibuprofen, avoiding the aggregation effects of prodrugs and improving the concentration of ibuprofen in the brain. As the above studies i*n vitro* demonstrated that the prodrugs possessed favorable physicochemical properties, we proceeded with the neuroprotective effect studies *in vitro* and *in vivo*.

### Cytotoxicity

**Figure 2.**
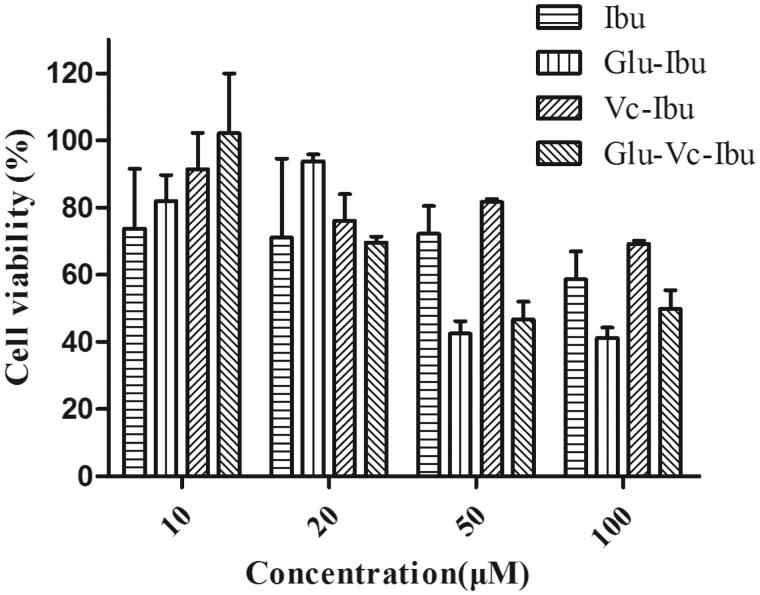
Cell viability upon addition of prodrugs in PC12 cells. Cell viability was expressed as a percentage of the control cell culture. Values are represented as mean ± SD (*n* = 3).

Cytotoxicity is one of the most critical factors to be considered in selecting prodrugs for biomedical applications. The toxicity of the prodrugs against PC12 cells was evaluated by MTT assays and the results were shown in Figure 2. The cell viability was over than 70% after incubating for 24 h at the concentration of the pharmacodynamics evaluation (10 μM), indicating that the ibuprofen prodrugs have a lower impact on cell survival.

### Neuroprotective effect

**Figure 3.**
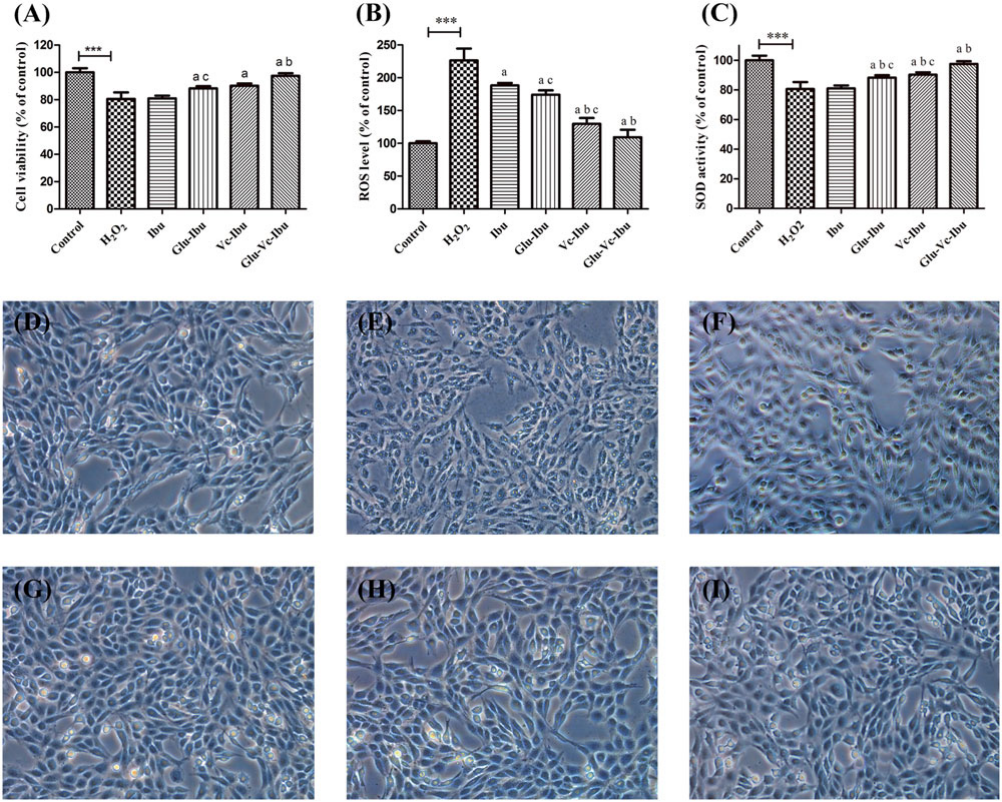
The neuroprotective effect of ibuprofen and the prodrugs. (A) Cell viability, (B) ROS level of ibuprofen and the prodrugs groups after H_2_O_2_ treatment *in vitro*, (C) SOD activity of ibuprofen and the prodrugs groups after H_2_O_2_ treatment *in vitro*. Cell morphology (D–I) in PC12 cells under hydrogen peroxide condition, (D) untreated PC12 cell, (E) PC12 cell exposed to H_2_O_2_ for 2 h, (F) PC12 cells pro-incubated with ibuprofen for 4 h and exposed to H_2_O_2_ for 2 h, (G) PC12 cells pro-incubated with Glu-Ibu for 4 h and exposed to H_2_O_2_ for 2 h, (H) PC12 cells pro-incubated with Vc-Ibu for 4 h and exposed to H_2_O_2_ for 2 h, (F) PC12 cells pro-incubated with Glu-Vc-Ibu for 4 h and exposed to H_2_O_2_ for 2 h. ***p < .001, a indicates significance at *p* < .05 versus H_2_O_2_ group, b indicates significance at *p* < .05 versus ibuprofen group, c indicates significance at *p* < .05 between Glu-Ibu or Vc-Ibu group and Glu-Vc-Ibu group. Data represent mean ± SD (*n* = 3).

The neuroprotective effect of ibuprofen and prodrugs was measured on a H_2_O_2_-induced injury model. Cell viability and morphological alteration of PC12 cells were shown in Figure 3. As shown in Figure 3(A), the H_2_O_2_-induced damage model was established successfully with a 66.64% survival rate compared to control. However, pretreatment with ibuprofen slightly relieved the damage (viability 74.10%). What’s more, the three prodrugs significantly relieved this damage and improved the viability of H_2_O_2_-treated cells (Glu-Ibu 80.56%, Vc-Ibu 81.92%, Glu-Vc-Ibu 89.91%), which indicated that the dual-targeting prodrug Glu-Vc-Ibu showed a significant efficacy compared with others (*p* < .05). Generation of intracellular ROS is another mechanism of oxidative damage to PC12 cells. It could be seen from Figure 3(B) that the ROS was overproduced (226.31% of control group) when PC12 cells was exposed to 1 μM H_2_O_2_. Pretreatment with the prodrugs significantly counteracted the ROS generated by H_2_O_2_, showing the better antioxidant effect than that of ibuprofen. What’s more, SOD is antioxidant defense system in cells to prevent ROS damage. Therefore, the activity of SOD was measured to evaluate the neuroprotective effect of ibuprofen and the prodrugs (Figure 3(C)). The results indicated that the SOD activity (80.58%) of PC12 cells was markedly decreased after treatment with H_2_O_2_ compared with the control group. While, the pretreatment with ibuprofen prodrugs resulted in the enhanced activities of SOD, even attempting to revert it to baseline. Noteworthy, the prodrug Glu-Vc-Ibu markedly relieved the damage compared with others (*p* < .05).

The morphological alteration of PC12 cells was observed using inverted phase-contrast microscopy shown in Figure 3(D–I). It could be seen from Figure 3(D) that the PC12 cell (control group) grew well with an intact morphology. But, the treatment of H_2_O_2_ disrupted the dendritic networks and markedly decreased the cell viability (Figure 3(E)). However, the oxidant damage was ameliorated with varying degrees by pretreatment with ibuprofen prodrugs (Figure 3(F–I)). As the above studies demonstrated that the prodrug Glu-Vc-Ibu possessed of superior activity *in vitro* compared with others, which might be ascribed to its dual brain-targeting ability, we proceeded with the brain ischemic injury model to further study the efficacy *in vivo*.

### Brain ischemia model

**Figure 4.**
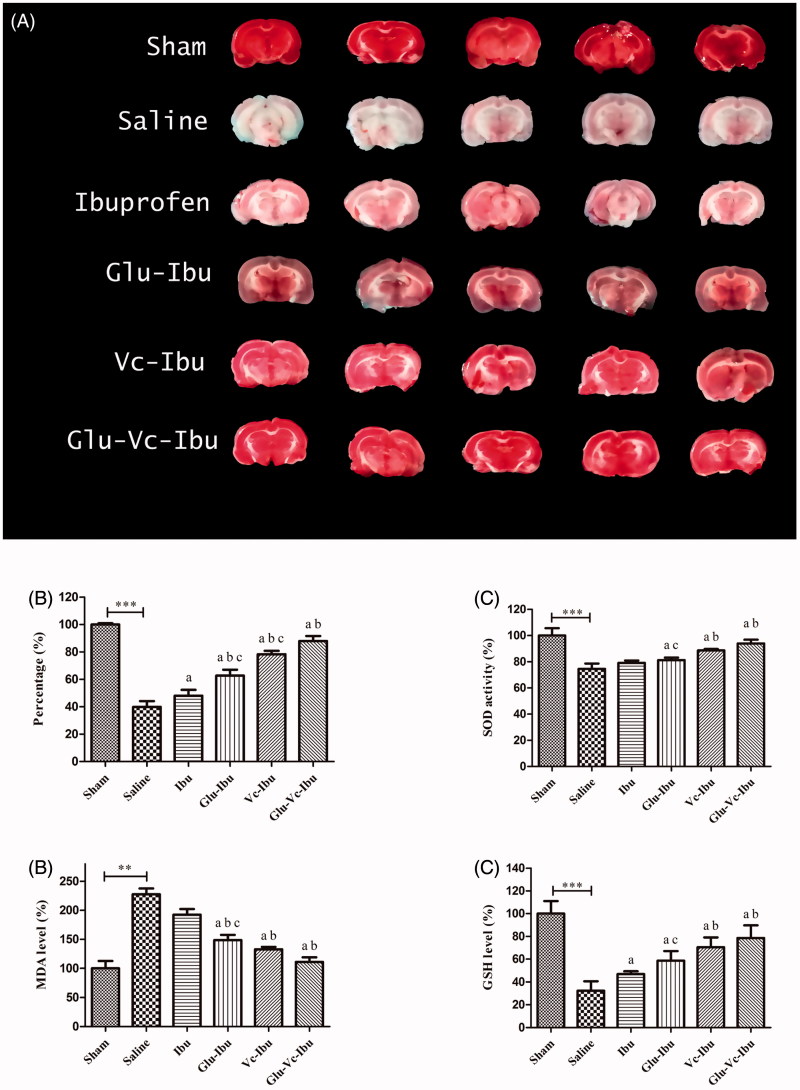
The neuroprotective effect on brain ischemia model. (A) TTC staining of the brain sections, (B) percentage of TTC staining rate compared with the sham group, (C) SOD activity, (D) MDA level, (E) GSH level. **p < .01, ***p < 0.001, a indicates significance at *p* < .05 versus saline group, b indicates significance at *p* < .05 versus ibuprofen group, c indicates significance at *p* < .05 between Glu-Ibu or Vc-Ibu group and Glu-Vc-Ibu group. Data are presented as mean ± SD (*n* = 5).

In the *in vivo* study, the brain ischemic injury model was established by ligating bilateral carotid arteries. TTC staining is commonly used to quantify experimental tissue infarctions. TTC, a marker of mitochondrial oxidative enzyme function, can accept a proton system and reduce itself to a red insoluble formazan product in normal brain sections. As shown in Figure 4(A), five brain sections of saline group, almost pale, indicated that the ischemia models was successfully established with serious brain damage. The increased neuroprotective function by administration of ibuprofen or these prodrugs could be seen from Figure 4(A) with the red-staining rate compared with the saline group. What’s more, the prodrug Glu-Vc-Ibu had the optimal therapeutic effect with the highest red-staining rate (87.95%) compared with the other treatment groups (ibuprofen 48.09%, Glu-Ibu 62.76%, Vc-Ibu 78.39%) shown in Figure 4(B).

SOD is one of the most important anti-oxidases. MDA, a degradation product of lipid peroxidation, has been widely studied as an index of cell membrane damage. GSH is a substrate of glutathione peroxidase, which could scavenge ROS *in vivo*. In this study, we used SOD, MDA and GSH as antioxidant markers. As shown in Figure 4(C), the ibuprofen administration did not obviously reverse the decrease of SOD activity in the ischemia injury animals (74.56% of the sham group). Though not significant different with ibuprofen, the SOD activity of prodrug Glu-Ibu group was significantly higher than saline group. Moreover, the SOD activity of Vc-Ibu and Glu-Vc-Ibu groups had significant raise compared with saline group or ibuprofen group, which indicated their delighted neuroprotective function. The MDA level of ischemia rats (saline 227.63%) was almost twice than that in the sham group, while ibuprofen or the prodrugs could suppress the increase in the MDA content caused by the injury with cerebral ischemia shown in Figure 4(D) (ibuprofen 192.42%, Glu-Ibu 148.59%, Vc-Ibu 132.61%, Glu-Vc-Ibu 110.96%). On the contrary, the levels of GSH in administration groups were significantly increased than that in the saline group (Figure 4(E)). At TTC staining, SOD activity, MDA and GSH level, the results indicated that these prodrugs, especially Glu-Vc-Ibu, had better neuroprotective function than that of other groups. Moreover, the results also suggested that the glucose–vitamin C system (Glu-Vc) could act as a vector to enhance the delivery of CNS drugs into brain. Given it efficiency and ease of synthesis, this approach may be applied in design of other brain-targeting drugs.

### Distribution in brain and pharmacokinetic studies in mice

**Figure 5.**
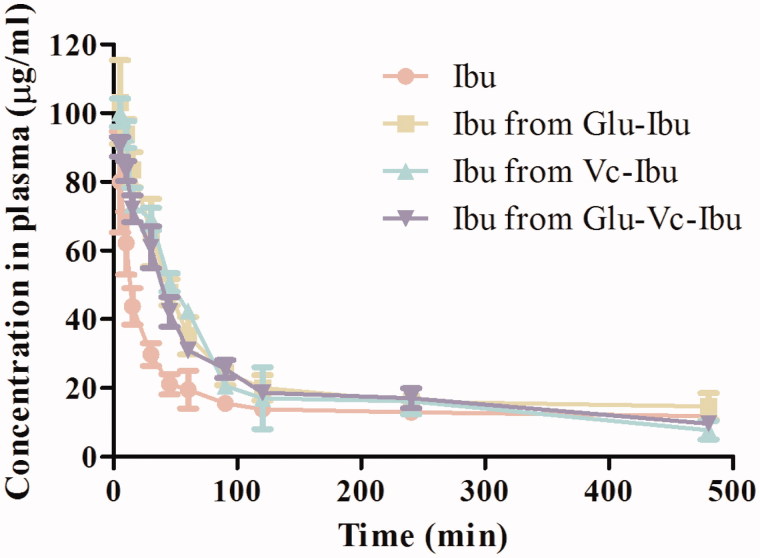
Concentration curves in plasma versus time after administration of Ibu, Glu-Ibu, Vc-Ibu and Glu-Vc-Ibu (hydrolysis, *n* = 3).

The curves in Figure 5 showed that the ibuprofen concentrations in blood from these prodrugs were higher than that of the direct ibuprofen administration. The concentrations of the prodrugs also declined much slower (Table 3). Because the prodrugs were more stable in plasma and had lower degradation rates, they had higher chances to be transported across the BBB into brain. The concentration curves of the prodrugs and released ibuprofen in plasma versus time after administration were shown in Supplementary Figure S1, and the pharmacokinetic parameters were listed in Supplementary Table S1. The concentration of ibuprofen was higher than that of the prodrugs groups (Supplementary Figure S1), which was ascribed to the stability of these prodrugs in plasma. Hence, the prodrugs had enough time to reach the brain before they were metabolized.

**Table 3.** Pharmacokinetic parameters in plasma (hydrolysis, *n* = 3).

Parameters	Ibuprofen	Ibu from Glu-Ibu	Ibu from Vc-Ibu	Ibu from Glu-Vc-Ibu
AUC_(0–_*_t_*_)_ (µg/ml min)	8250.62 ± 412.86	11607.51 ± 354.13	10,495.56 ± 438.25	10,483.82 ± 591.91
MRT (min)	65.47 ± 3.12	92.18 ± 3.74	88.33 ± 5.38	99.46 ± 2.55
*T*_max_ (min)	5	5	5	5
*C*_max_ (µg/ml)	80.03 ± 12.41	103.24 ± 13.45	100.15 ± 8.34	90.17 ± 7.69

**Figure 6.**
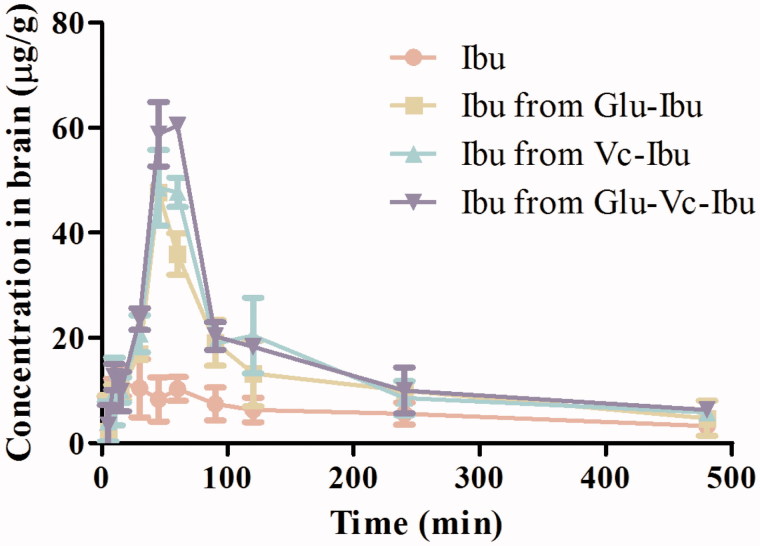
Concentration curves in brain homogenate versus time after administration of Ibu, Glu-Ibu, Vc-Ibu and Glu-Vc-Ibu (hydrolysis, *n* = 3).

The *in vivo* pharmacokinetic evaluation of prodrugs gives new insight into the possibility of utilizing glucose and Vc as means for brain targeting. It is obvious that the prodrugs could be delivered to the brain after intravenously administration (Figure 6). The concentration of ibuprofen was much higher when prodrugs were administrated than when naked ibuprofen was used at any interval during 8 h. The AUC_0–_*_t_* and *C*_max_ of ibuprofen for these prodrugs were fairly higher than that of naked ibuprofen (Table 4). The relative uptake efficiencies (REs) were enhanced for ibuprofen from prodrugs Glu-Ibu, Vc-Ibu and Glu-Vc-Ibu, for which the values were 2.10, 2.38 and 2.63 times higher, respectively, than that of the naked ibuprofen. The concentration efficiencies (CEs) were also increased to 4.13, 4.20 and 5.24 times higher than that of ibuprofen. More importantly, prodrug Glu-Vc-Ibu was able to penetrate the BBB and release ibuprofen in the brain *in vivo* (Supplementary Figure S2). The AUC_0–_*_t_* and *C*_max_ of Glu-Vc-Ibu in brain were obviously higher than that of the parent drug (Supplementary Table S2), although the opposite trend was seen in plasma. What’s more, the brain concentration of ibuprofen released from Glu-Vc-Ibu was approximately three times of that found in brain after ibuprofen was administrated for 45 min, which suggested that the prodrugs might have a slow release, long-term effect. These data further indicated our design was feasible, that the prodrug Glu-Vc-Ibu could maintain sufficient levels and deliver ibuprofen into the brain.

**Table 4.** Pharmacokinetic parameters of ibuprofen in brain homogenate (hydrolysis, *n* = 3).

Parameters	Ibuprofen	Ibu from Glu-Ibu	Ibu from Vc-Ibu	Ibu from Glu-Vc-Ibu
AUC_(0–_*_t_*_)_ (µg/g min)	2782.42 ± 176.55	5859.63 ± 337.28	6629.24 ± 467.29	7308.11 ± 447.86
MRT (min)	190.28 ± 15.11	164.08 ± 20.52	158.37 ± 9.33	156.77 ± 13.09
*T*_max_ (min)	15	45	45	50
*C*_max_ (µg/g)	11.54 ± 1.49	47.69 ± 5.77	48.54 ± 6.34	60.43 ± 2.37
RE	–	2.10	2.38	2.63
CE	–	4.13	4.20	5.24

## Conclusion

In order to develop an efficient brain-targeting drug delivery system to greatly improve the brain permeability of the drug, we used ibuprofen as a model parent drug, designed, synthesized and evaluated a dual-targeting prodrug co-modified by glucose and Vc. The Glu-Vc moiety significantly increased the brain uptake of ibuprofen *in vivo* with the improved neuroprotective effect compared with naked ibuprofen or other singly-modified prodrugs, suggesting that both glucose and Vc transporters (GLUT_1_ and SVCT_2_) might be involved to help the prodrug to penetrate the BBB. Therefore, the Glu-Vc modification represents a promising strategy for the development of future brain-specific drug delivery systems.

## Supplementary Material

IDRD_Wu_et_al_Supplemental_Content.doc
