# Supplementary material for: Dual-targeting for brain-specific drug delivery: synthesis and biological evaluation
Source: Drug Deliv. 2018 Jan 31;25(1):426–34. doi: 10.1080/10717544.2018.1431978 (PMC6058731; doi:10.1080/10717544.2018.1431978)
Supplement: IDRD_Wu_et_al_Supplemental_Content.doc [file IDRD_A_1431978_SM8805.doc]

**Dual-targeting for brain-specific drug delivery: synthesis and biological evaluation**

Qiming Yue^1^, Yao Peng^1^, Yi Zhao, Runxin Lu, Qiuyi Fu, Yang Chen, Yang Yang,

Li Hai, Li Guo*, Yong Wu*

*Key Laboratory of Drug Targeting and Drug Delivery System of Education Ministry, Department of Medicinal Chemistry, West China School of Pharmacy, Sichuan University, Chengdu 610041, P. R. of China*

^1^These authors contributed equally to this work.

*Corresponding authors. guoli@scu.edu.cn (L. Guo), wyong@scu.edu.cn (Y. Wu).

***Supporting Information***

Synthesis of prodrug Glu-Ibu ······························································P2-P3

Synthesis of prodrug Vc-Ibu····································································P4

Figure S1··························································································P5

Table S1···························································································P6

Figure S2··························································································P7

Table S2···························································································P8

^1^H NMR of the compounds································································P9-P11

References························································································P12

**Synthesis of prodrug Glu-Ibu**

Synthesis of prodrug Glu-Ibu. Reagents and conditions: (a) ibuprofen, DCC, DMAP, CH_2_Cl_2_, r.t.; (b) Pd/C, H_2_, CH_3_OH, r.t.

*Synthesis of compound* ***12***

To a solution of ibuprofen (0.12 g, 0.58 mmol) in CH_2_Cl_2_ (10 ml) was added DCC (0.16 g, 0.77 mmol) and DMAP (10 mg, 0.07 mmol), and the reaction was stirred at -5 ^o^C for 30 min. Then compound **4** (0.21 g, 0.38 mmol) in CH_2_Cl_2_ (5 ml) was added dropwise. After stirring for another 20 h at room temperature, the mixture was filtered and the filtrate was concentrated. Then the residue was purified by chromatography to give **12** (0.23 g, 82%) as a white solid. ^1^H NMR (400 MHz, CDCl_3_) *δ*: 0.77-0.88 (m, 6H), 1.50 (t, 3H, *J* = 6.8 Hz), 1.75-1.82 (m, 1H), 2.28-2.45 (m, 2H), 3.36-3.50 (m, 3H), 3.60 (q, 1H, *J* = 8.8 Hz), 3.71-3.80 (m, 1H), 4.16-4.20 (m, 1H), 4.41-4.47 (m, 2H), 4.51-4.59 (m, 2H), 4.68-4.78 (m, 3H), 4.83-4.96 (m, 3H), 6.98-7.34 (m, 24H). HRMS: (ESI+) calculated for C_47_H_52_O_7_Na [M+Na]^+^ 751.3611, found 751.3614. Elemental Analysis: C, 77.45; H, 7.19, found C, 77.36; H, 7.11.

*Synthesis of prodrug Glu-Ibu*

To a solution of compound **12** (0.10 g, 0.14 mmol) in methanol, Pd/C (10 mg, 10%) was added. Then, the mixture was stirred in hydrogen atmosphere at room temperature for 6 h. Pd/C was filtered, and the filtrate was concentrated to give Glu-Ibu (46 mg, 92.0%) as a white solid. ^1^H NMR (400 MHz, CD_3_OD) *δ*: 0.85 (d, 6H, *J* = 6.4 Hz), 1.42 (s, 3H), 1.78-181 (m, 1H), 2.45 (s, 2H), 3.44-4.42 (m, 11H), 7.04 (s, 2H), 7.15 (s, 2H). HRMS: (ESI+) calculated for C_19_H_28_O_7_Na [M+Na]^+^ 391.1733, found 391.1730. Elemental Analysis: C, 61.94; H, 7.66, found C, 61.90; H, 7.61.

**Synthesis of prodrug Vc-Ibu**

Synthesis of prodrug Vc-Ibu. Reagents and conditions: (a) TrtCl, Et_3_N, CH_2_Cl_2_, r.t.; (b) DCC, DMAP, overnight; (c) HCl, CH_3_CN, 50 ^o^C; (d) Pd/C, H_2_, CH_3_OH, r.t.

The synthesis of prodrug Vc-Ibu was reported in our previous work (Zhao et al., 2014).


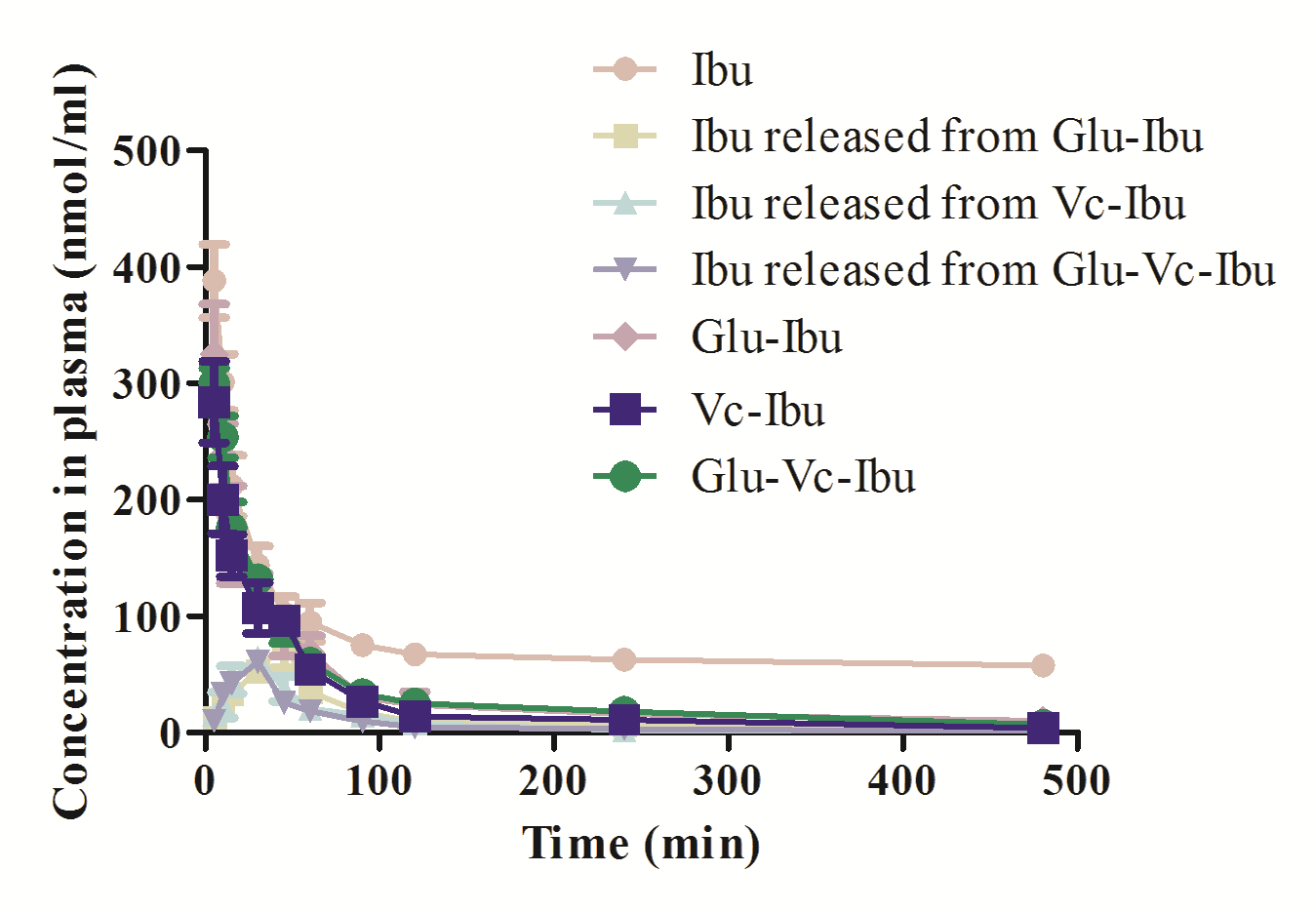


**Figure S1:** Concentration curves in plasma versus time after administration of ibuprofen (Ibu), Glu-Vc-Ibu, Glu-Ibu, Vc-Ibu. (n=3).

**Table S1:** Pharmacokinetic parameters of ibuprofen and prodrugs in plasma. (n=3)

| Parameters | AUC_(0-t)_ (µg/ml·min) | MRT (min) | T_max_ (min) | C_max_ (µg/ml) |
| --- | --- | --- | --- | --- |
| Ibuprofen | 8250.62 ± 412.86 | 65.47 ± 3.12 | 5 | 80.03 ± 12.41 |
| Ibu released from Glu-Ibu | 1307.93 ± 112.51 | 85.16 ± 4.39 | 45 | 16.05 ± 2.02 |
| Ibu released from Vc-Ibu | 863.55 ± 67.14 | 81.01 ± 6.99 | 30 | 13.24 ± 0.96 |
| Ibu released from Glu-Vc-Ibu | 758.42 ± 42.37 | 90.90 ± 8.65 | 30 | 12.56 ± 1.46 |
| Glu-Ibu | 3513.41 ± 257.33 | 106.48 ± 11.86 | 5 | 119.73 ± 8.79 |
| Vc-Ibu | 2779.75 ± 114.95 | 83.85 ± 4.49 | 5 | 103.48 ± 6.75 |
| Glu-Vc-Ibu | 3531.33 ± 136.03 | 101.65 ± 8.28 | 5 | 187.98 ± 15.46 |


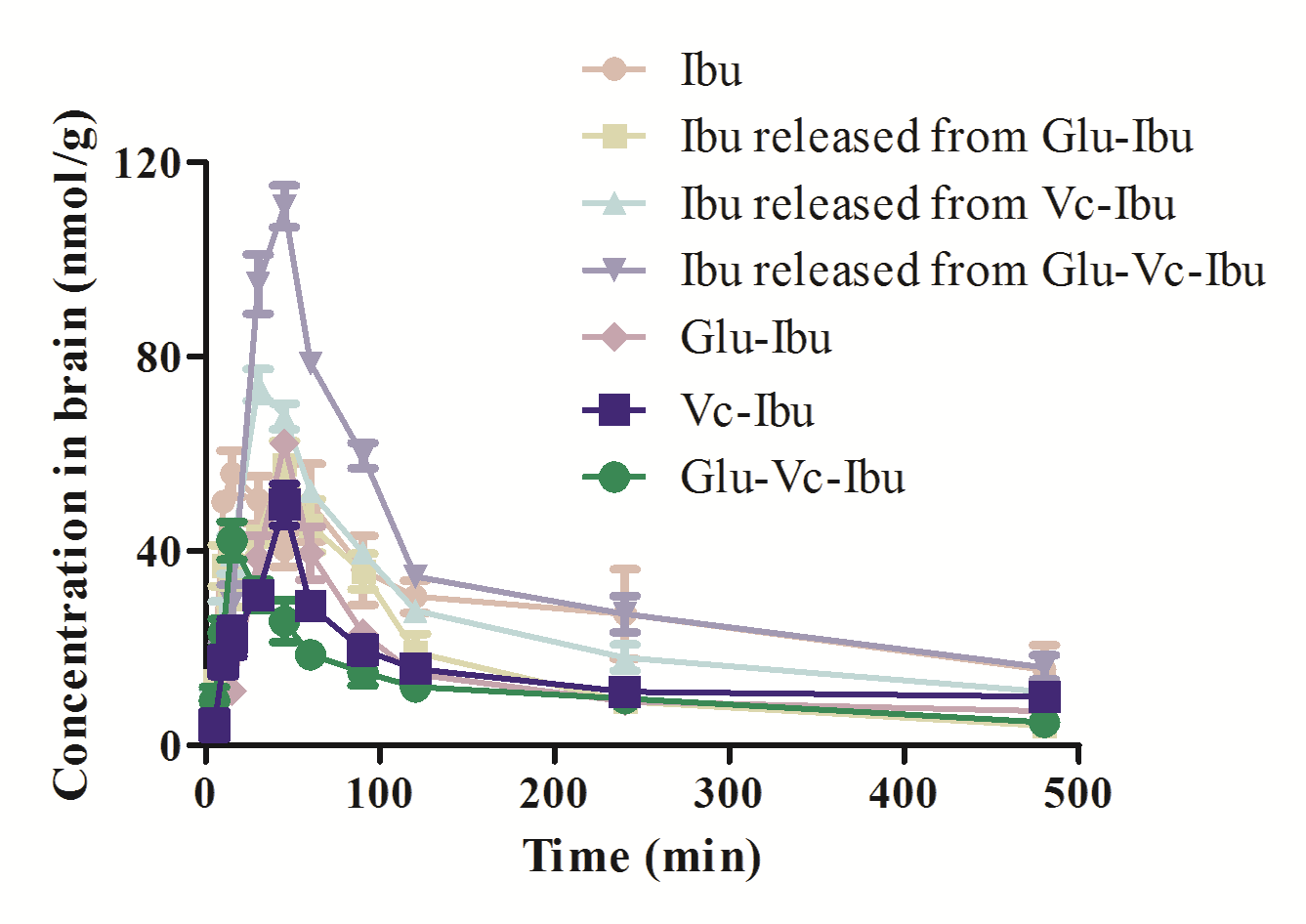


**Figure S2:** Concentration curves in brain versus time after administration of ibuprofen (Ibu), Glu-Vc-Ibu, Glu-Ibu, Vc-Ibu. (n=3).

**Table S2: Pharmacokinetic parameters of ibuprofen and prodrugs in brain. (n=3)**

| Parameters | AUC_(0-t)_ (µg/g·min) | MRT (min) | T_max_ (min) | C_max_ (µg/g) |
| --- | --- | --- | --- | --- |
| Ibuprofen | 2782.42 ± 176.55 | 190.28 ± 15.11 | 15 | 11.54 ± 1.49 |
| Ibu released from Glu-Ibu | 2099.55 ± 156.57 | 130.88 ± 9.65 | 45 | 11.92 ± 5.94 |
| Ibu released from Vc-Ibu | 2412.74 ± 202.38 | 164.58 ± 13.24 | 30 | 15.30 ± 0.95 |
| Ibu released from Glu-Vc-Ibu | 3393.98 ± 257.39 | 169.61 ± 16.91 | 45 | 22.87 ± 3.76 |
| Glu-Ibu | 2520.48 ± 115.82 | 236.98 ± 23.02 | 45 | 22.90 ± 4.29 |
| Vc-Ibu | 2601.14 ± 98.07 | 187.23 ± 8.37 | 45 | 18.05 ± 1.65 |
| Glu-Vc-Ibu | 3459.18 ± 223.76 | 163.72 ± 11.39 | 15 | 26.40 ± 1.97 |

**^1^H NMR of Compound 10**


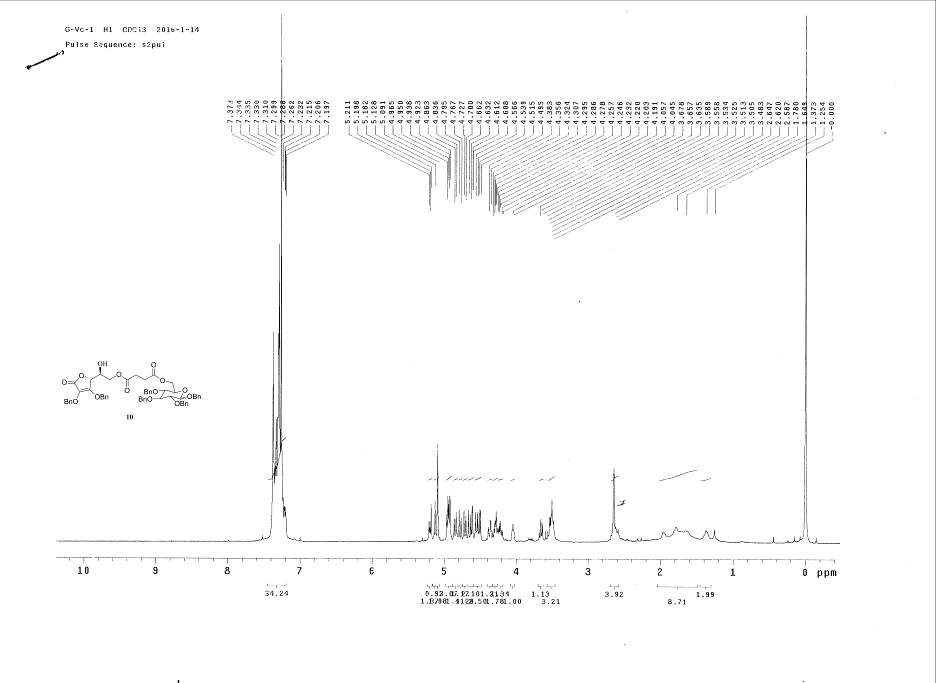


**^1^H NMR of Compound 11**


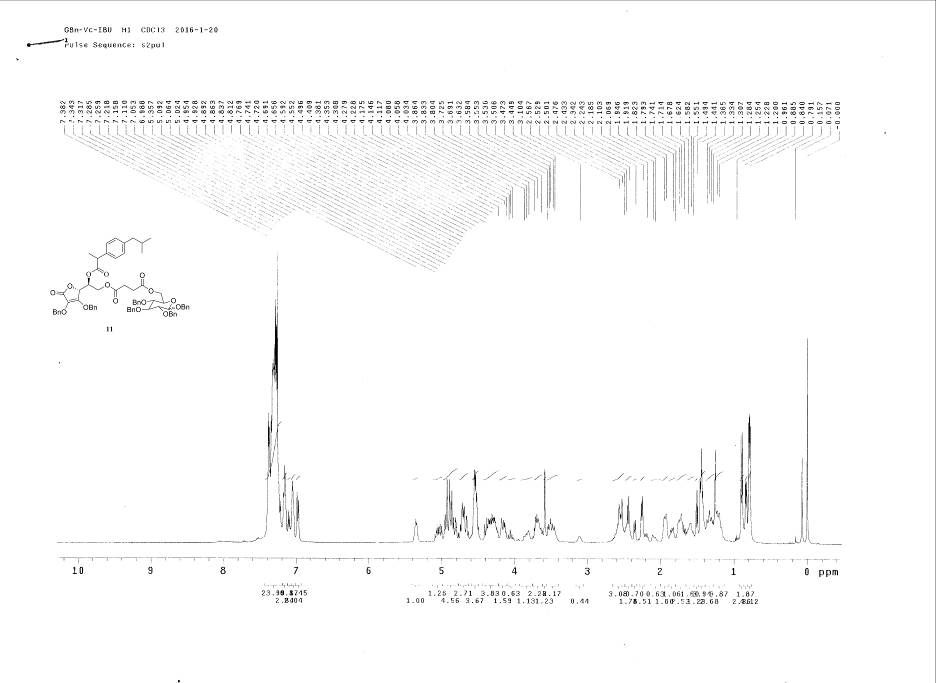


**^1^H NMR of prodrug Glu-Vc-Ibu**


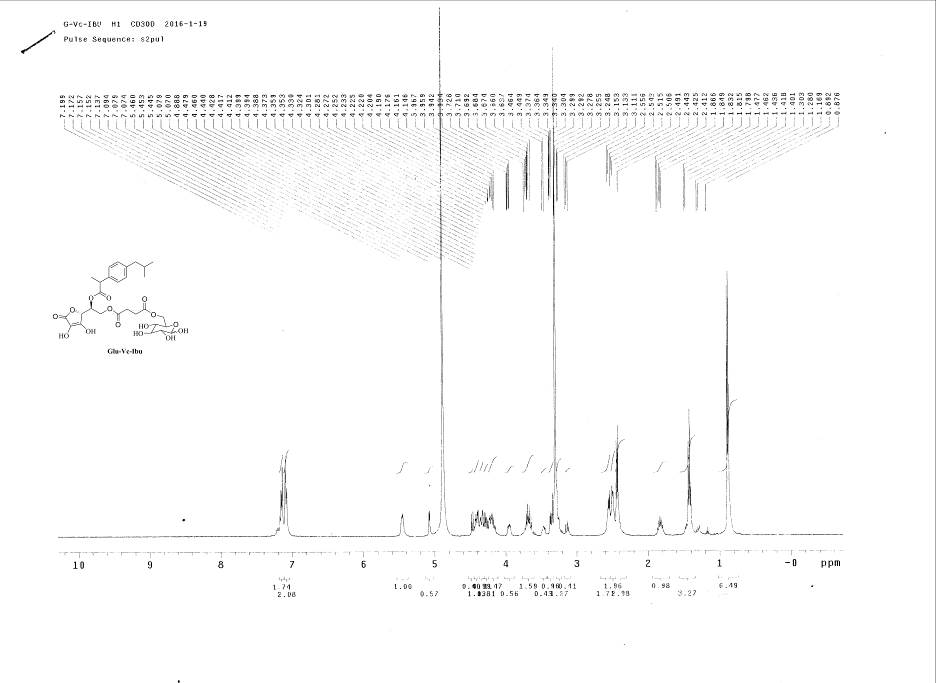


**^1^H NMR of Compound 12**


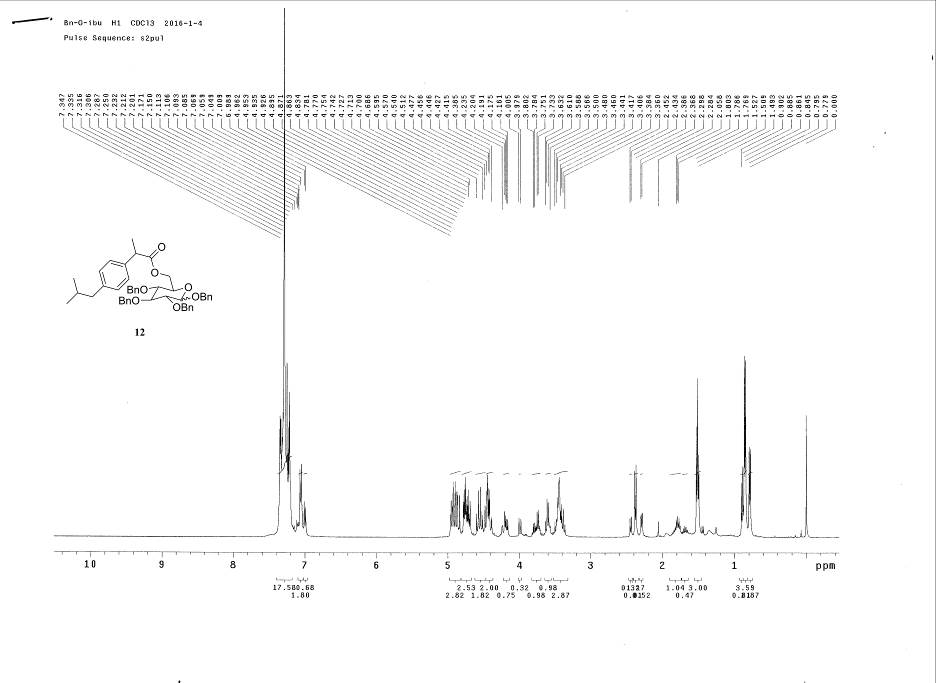


**^1^H NMR of prodrug Glu-Ibu**


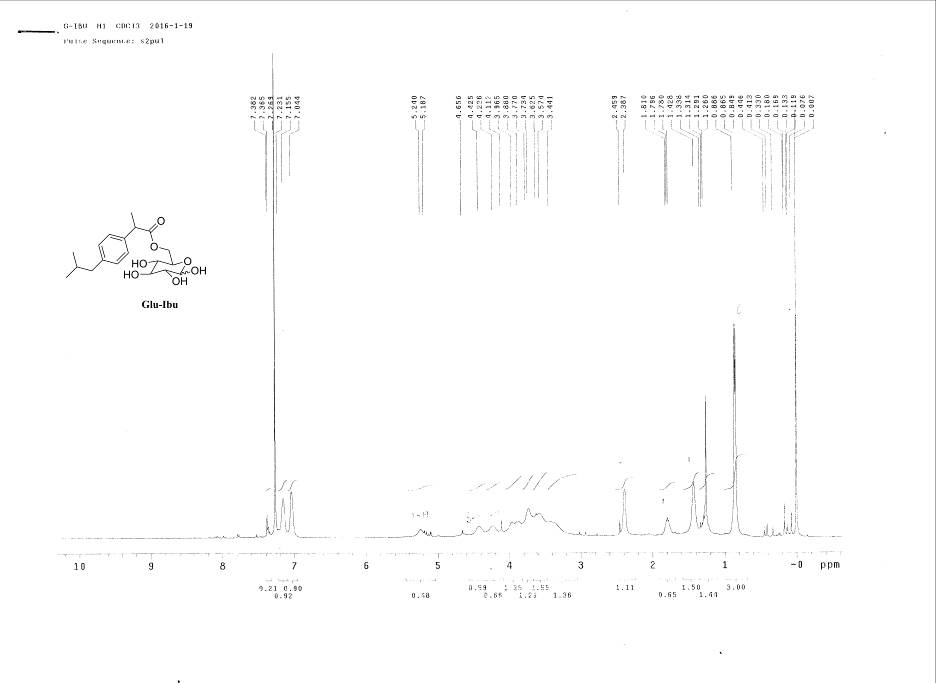


**References**

Zhao Y, Qu BY, Wu XY, et al. (2014). Design, synthesis and biological evaluation of brain targeting L-ascorbic acid prodrugs of ibuprofen with "lock-in" function. Eur J Med Chem 82:314-323.
